# Generating orthogonal glycosyltransferase and nucleotide sugar pairs as next-generation glycobiology tools

**DOI:** 10.1016/j.cbpa.2020.09.001

**Published:** 2021-02

**Authors:** Anna Cioce, Stacy A. Malaker, Benjamin Schumann

**Affiliations:** 1 Chemical Glycobiology Laboratory, The Francis Crick Institute, 1 Midland Road, NW1 1AT, London, United Kingdom; 2 Department of Chemistry, Imperial College London, 80 Wood Lane, W12 0BZ, London, United Kingdom; 3 Department of Chemistry, Stanford University, 290 Jane Stanford Way, Stanford, CA, 94305, USA; 4 Department of Chemistry, Yale University, 275 Prospect Street, New Haven, CT, 06511, USA

**Keywords:** Glycosylation, Bioorthogonal, Glycosyltransferase, Mucin, Glycoprotein, Protein engineering, Click chemistry

## Abstract

Protein glycosylation fundamentally impacts biological processes. Nontemplated biosynthesis introduces unparalleled complexity into glycans that needs tools to understand their roles in physiology. The era of quantitative biology is a great opportunity to unravel these roles, especially by mass spectrometry glycoproteomics. However, with high sensitivity come stringent requirements on tool specificity. Bioorthogonal metabolic labeling reagents have been fundamental to studying the cell surface glycoproteome but typically enter a range of different glycans and are thus of limited specificity. Here, we discuss the generation of metabolic ‘precision tools’ to study particular subtypes of the glycome. A chemical biology tactic termed bump-and-hole engineering generates mutant glycosyltransferases that specifically accommodate bioorthogonal monosaccharides as an enabling technique of glycobiology. We review the groundbreaking discoveries that have led to applying the tactic in the living cell and the implications in the context of current developments in mass spectrometry glycoproteomics.

## Introduction

**Figure 1:**
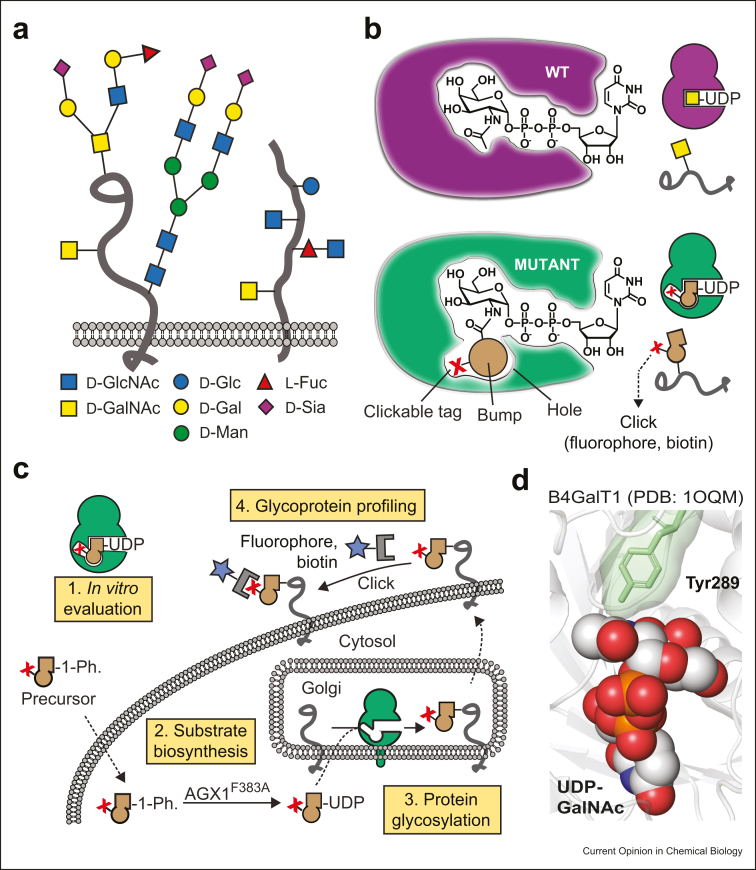
**Using glycosyltransferase bump-and-hole engineering to understand cell surface glycosylation. (a)** Diversity of cell surface glycans. (**b)** Bump-and-hole engineering enlarges the active site of a GT to accommodate a chemically modified nucleotide-sugar containing a bioorthogonal tag. (**c)** A blueprint of key steps to establish a cellular GT bump-and-hole system [11]. (**d)** Co-crystal structure of WT-B4GALT1 (PDB 1OQM) with UDP-GalNAc. B4GALT1 was subsequently engineered to accommodate bioorthogonal UDP-GalNAc analogs [25]. Adapted from *Molecular Cell,* Vol 78/5, B. Schumann et al., Bump-and-Hole Engineering Identifies Specific Substrates of Glycosyltransferases in Living Cells, 824–834.e15, Copyright (2020), with permission from Elsevier. GTs, glycosyltransferases.

Protein glycosylation is the most complex post-translational modification. Dysfunctions in the biosynthesis and turnover of glycan structures (the ‘glycome’), as well as underlying glycoproteins (the ‘glycoproteome’) are associated with disease [1]. Attachment to proteins is primarily via Asn (N-linked) or Ser/Thr/Tyr (O-linked) side chains. Although N-linked glycosylation is found on consensus peptide sequons (N_X_S/T) and can thus be predicted, O-linked glycosylation often lacks such sequons (Figure 1a) [2,3]. Cell surface glycans are biosynthesized from 10 monosaccharide units by the combinatorial activity of more than 250 glycosyltransferases (GTs). Although the secretory pathway comprises an arsenal of GTs that influence each other through compensation and competition [4], deficiencies of individual GTs are related to congenital disorders of glycosylation [5]. Despite forays made into understanding the molecular details of GT activity, our insights are still limited by the complexity of the secretory pathway and the analytical challenges associated with studying glycans.

Glycoproteome analysis helps elucidate many structural and functional properties of glycoproteins as a readout of GT activity. These analyses have been fueled by tools in biology and chemistry. Standing out among the latter are the metabolic labeling (also called metabolic oligosaccharide engineering [MOE]) reagents that contain a bioorthogonal, reactive handle [6]. When fed to growing cells, the MOE reagent is incorporated into newly synthesized glycoproteins via biosynthetic machineries. Functionalized glycoproteins are then reacted with an enrichment probe allowing isolation and MS analysis [7], or a fluorescent probe for or imaging in living cells [8,9]. MOE reagent and probe must possess complementary functional groups that are chemically inert in a biological environment while reactive toward each other. Most bioorthogonal reactions involve copper-assisted (CuAAC) or strain-promoted (SPAAC) azide–alkyne cycloaddition, inverse electron demand Diels–Alder reactions between strained or terminal alkenes and tetrazine reporters [10], and the Staudinger ligation between azides and phosphines [6,8].

MOE reagents have produced valuable insights into various aspects of glycobiology [11∗∗, 12∗∗, 13]. Nevertheless, these reagents are of limited specificity by default for two reasons: (i) interconversion into other monosaccharides with different biosynthetic fates and (ii) the substrate promiscuity of certain GTs. Thus, the bioorthogonal label can be incorporated into undesired substructures within the glycome. With the advent of sensitive methods of quantitative biology, more specific tools are needed to inform on the products of individual GTs [14].

Studying the products of individual members of a transferase family is the prime discipline of a chemical biology tactic called ‘bump-and-hole (BH) engineering’. In a structure-guided process, an enzyme's catalytic pocket is enlarged by mutating bulky ‘gatekeeper’ amino acids into smaller ones, to create a BH mutant. This strategy creates an often hydrophobic ‘hole’ in the active site that is complementary to a synthetic substrate containing a bulky, ‘bumped’ functional group (Figure 1b). As the bumped substrate is only recognized by the BH mutant, this new enzyme–substrate pair is orthogonal to all other transferases in a complex system such as living cells. After inception in the field of kinases by Alaimo et al. Shokat and colleagues, [15], successful application to several enzyme families [16, 17, 18, 19, 20, 21, 22, 23, 24] has laid the foundation to the tactic being applied to GTs as an enabling method in the glycosciences (Figure 1c).

### The early days of GT BH engineering

In the early 2000s, Qasba and Ramakrishnan [25] engineered a GT to accommodate nonnatural, bioorthogonal uridine diphosphate(UDP)-sugars for the first time. Bovine β-1,4-galactosyltransferase (B4GalT1) normally transfers galactose to *N*-acetylglucosamine (GlcNAc)-terminating structures. Mutation of the residue Tyr298 to Leu rendered BH-B4GalT1 reactive toward UDP-*N*-acetylgalactosamine (UDP-GalNAc) derivatives such as UDP-2-keto-galactose and UDP-azido-*N*-acetylgalactosamine **1** (UDP-GalNAz) (Figure 1d) [26, 27, 28]. These analogs are not bulky enough to be considered ‘bumped’, as they are used by many other WT-GTs [14,29]. Moreover, the cytosolic UDP-GalNAc/GlcNAc 4′-epimerase GALE interconverts UDP-GalNAz into the corresponding UDP-GlcNAc derivative, UDP-GlcNAz [14]. BH-B4GalT1/UDP-GalNAz would thus not be amenable for use in the living cell. Nevertheless, through the efforts of Hsieh-Wilson and others, the BH-B4GalT1/UDP-GalNAz system has been developed into a tool to profile proteins with the nucleocytoplasmic Ser/Thr-linked O-GlcNAc modification *in vitro.* [30, 31, 32, 33∗, 34, 35].

### Reprogramming metabolism to deliver UDP-GalNAc analogs into living cells

**Figure 2:**
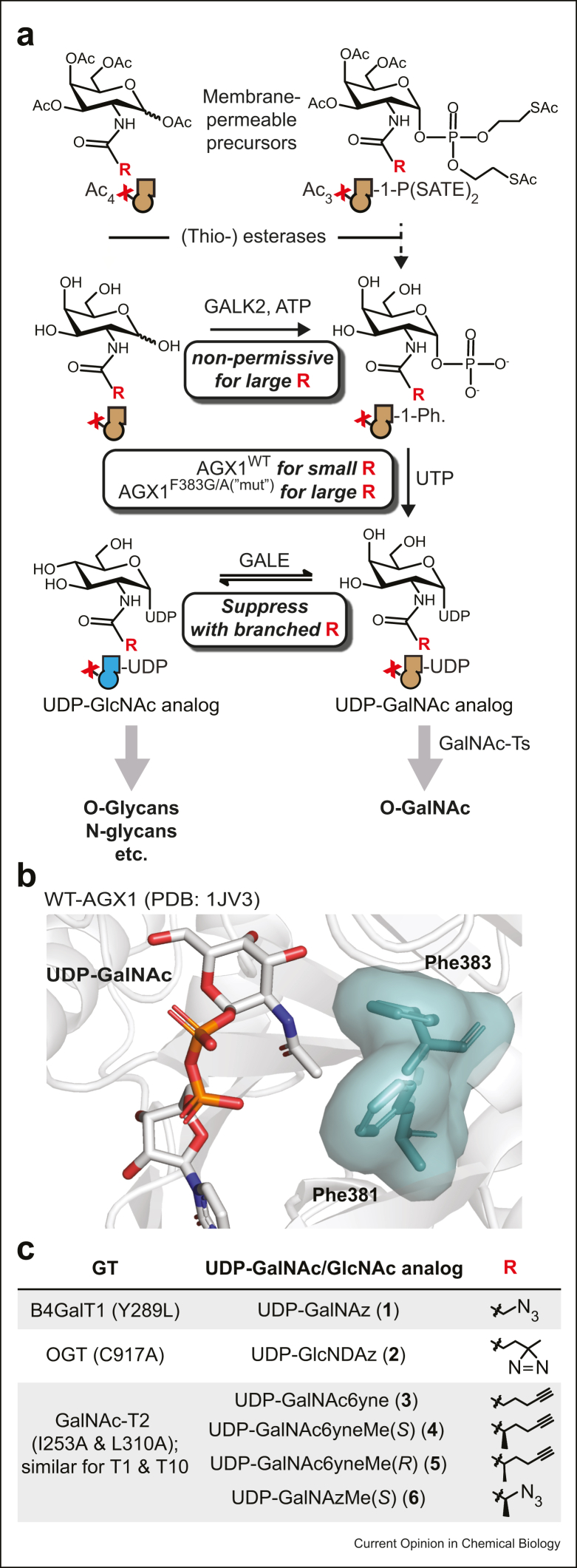
**Reprogramming metabolism to deliver UDP-sugar analogs. (a)** Schematic representation of the GalNAc salvage pathway applied to chemically modified GalNAc analogs. Suitable membrane-permeable precursors can be used to circumvent the GALK2 step if needed, but AGX1 engineering is necessary to deliver bumped UDP-GalNAc analogs. GALE-mediated epimerization to UDP-GlcNAc analogs can be suppressed by using branched acylamide side chains [12]. (**b)** Co-crystal structure of WT-AGX1 with UDP-GalNAc (PDB 1JV3) used to rationalize the F383G/A mutation that biosynthesizes bumped UDP-sugar analogs. (**c)** Structures of UDP-sugar analogs used in conjunction with GT engineering. GTs, glycosyltransferases.

BH approachA cellular GT bump-and-hole approach requires the biosynthesis of bumped UDP-sugars in the cytosol. Piller and colleagueset al. [36] found that the enzymes of the GalNAc salvage pathway – the kinase GALK2 and the pyrophosphorylase AGX1 – exhibit low promiscuity toward chemical modifications of the GalNAc acetamide (Figure 2a). Bumped GalNAc analogs are thus not effective substrates of either enzyme [11,12,37]. Because AGX1 and its close homolog AGX2 are also components of the GlcNAc salvage pathway, low substrate promiscuity impeded delivery of both bumped UDP-GalNAc and UDP-GlcNAc derivatives.

Again, protein engineering came to the rescue. Kohler et al. [37] mutated the gatekeeper residues Phe381 or Phe383 in human AGX1 to Gly and assessed turnover of the bumped GlcNDAz-1-phosphate (Figure 2b) [43]. The mutant AGX1^F383G^ was subsequently used to endow cells with the capacity to biosynthesize UDP-GlcNDAz **2** (Figure 2c). To circumvent the nonpermissive GALK2 step, a caged, membrane-permeable GlcNDAz-1-phosphate precursor was used for delivery. GlcNDAz was then used as a photo-crosslinkable reporter of O-GlcNAc, allowing for mapping of protein interaction partners by MS proteomics, eventually in conjunction with BH engineering of O-GlcNAc transferase OGT [38].

### BH engineering of a human GT family: polypeptide GalNAc transferases (GalNAc-Ts)

Attempts to BHbump-and-hole engineer an entire GT family started in the early 2000s (personal communication by C. R. Bertozzi), but took almost two decades to be brought to fruition. The polypeptide GalNAc transferases (GalNAc-Ts) constitute one of the largest GT families in the human genome. Approximately 20 isoenzymes (called T1-T20) catalyze the first step in the biosynthesis of glycans primed by GalNAcα1-*O*-Ser/Thr, also called O-GalNAc or mucin-type protein glycosylation. Although GalNAc-Ts have been connected to a wide variety of diseases [2,39, 40, 41, 42], it is still challenging to associate individual isoenzymes with a specific biological function due to their complex interplay in the secretory pathway. Uniquely suited to dissect GalNAc-T biology, the BH approach was not amenable to the GalNAc-T family before a series of key requirements were met by the groundbreaking work by Qasba, Hsieh-Wilson, Bertozzi, Kohler and many others.

**Figure 3:**
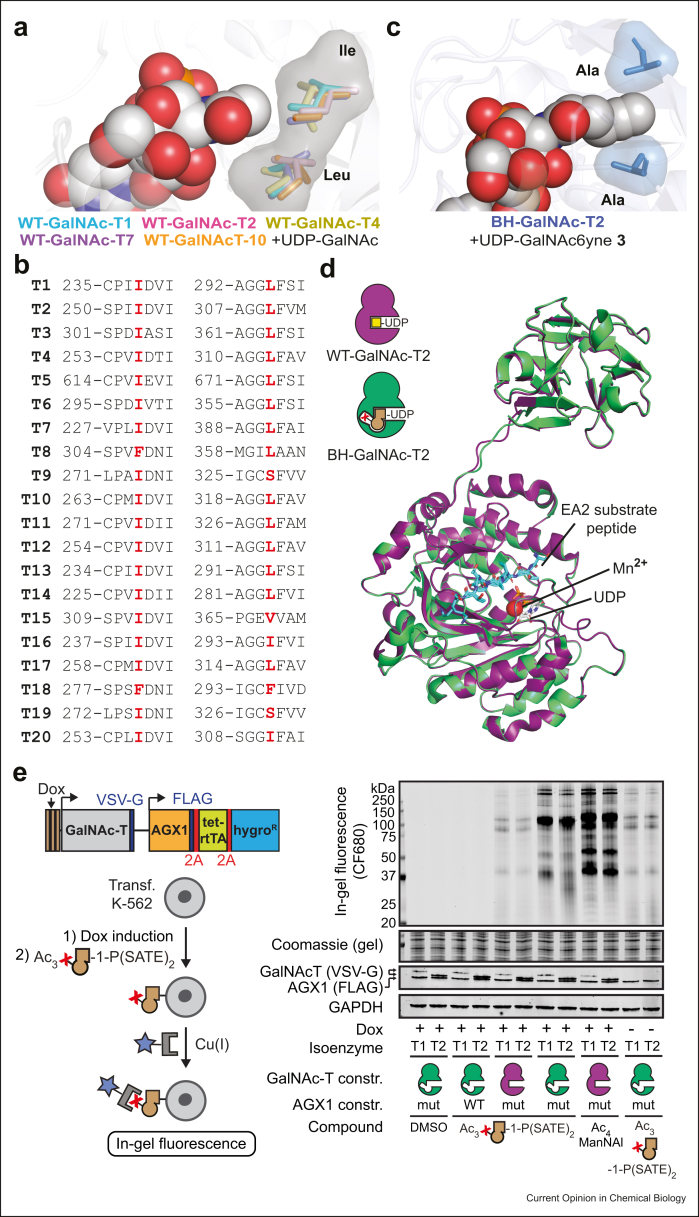
**Structural basis for GalNAc-T bump-and-hole engineering. (a)** Gatekeeper residues identified in the crystal structures of GalNAc-T1 (PDB 1XHB), T2 (PDB 4D0T), T4 (PDB 5NQA), T7 (PDB 6IWR), and T10 (PDB 2D7I). (**b)** Sequence alignment of gatekeeper residues in all 20 GalNAc-Ts. (**c)** Co-crystal structure of BH-GalNAc-T2 (PDB 6NQT) with UDP-GalNAc6yne **3**. Gatekeeper residues are mutated to Ala to accommodate the aliphatic alkyne. (**d)** Superposition of WT-GalNAc-T2 (PDB 2FFU) and BH-GalNAc-T2 (PDB 6E7I) with EA2 substrate peptide (overlay of both structures), Mn^2+^ and UDP. (**e)** Metabolic labeling of cells transfected with AGX1 (WT or mut) and either GalNAc-T1 or T2 (WT or BH-mutant) constructs. Dox-inducible GalNAc-T expression was used in conjunction with feeding a caged precursor of UDP-GalNac6yne **3**. DMSO and the tagged sialic acid precursor Ac_4_ManNAl served as negative and positive controls, respectively. Panel **E** reprinted from *Molecular Cell,* Vol 78/5, B. Schumann et al., Bump-and-Hole Engineering Identifies Specific Substrates of Glycosyltransferases in Living Cells, 824–834.e15, Copyright (2020), with permission from Elsevier.

The BH approach needs an enzyme/substrate co-crystal structure, the first of which (GalNAc-T10 and GalNAc) was published by Narimatsu and colleagueset al. [43]. The gatekeeper residues Ile and Leu could be identified and mapped for other isoenzymes based on structural and sequence homology (Figure 3a and b). Second, bioorthogonal, bumped UDP-GalNAc analogs were required to identify suitable BH-enzyme-substrate pairs *in vitro*. Choi, Wagner et al. [44] used a combination of chemical and chemoenzymatic syntheses to develop a collection of 20 UDP-GalNAc analogs. Mutation of both gatekeeper residues to Ala reprogrammed the nucleotide sugar specificity of WT-GalNAc-T1, T2, and T10 to chemically modified UDP-GalNAc analogs such as alkynes **3**–**5** *in vitro* (Figure 3a, c). We subsequently found that engineering preserved both the three-dimensional structure of T2 and peptide substrate preference of T1 and T2 [11], ascertaining WT-like behavior of BH-GalNAc-Ts (Figure 3d).

A GalNAc-T BH system was then assembled in living cells. Our approach featured AGX1-mediated substrate delivery based on Kohler's strategy. Using the mutant AGX1^F383A^, we delivered UDP-GalNAc analog **3** into living cells from the membrane-permeable, caged GalNAc-1-phosphate analog Ac_3_GalNAc6yne-1-P(SATE)_2_ that is deprotected in the cytosol, presumably by esterases and thioesterases (Figure 1, Figure 2a). Of note, the AGX1 F383A mutant had largely similar properties to the F383G mutant but accepted a broader GalNAc-1-phosphate substrate range in our hands and is called here ‘mut-AGX1’ (Figure 2a) [12]. Monitoring UDP-sugar biosynthesis by ion exchange chromatography of cell extracts was essential at this point to confirm substrate delivery. Immunofluorescence further revealed that BH-T1 and BH-T2 localize to the Golgi compartment, and expression can be controlled with a doxycycline (Dox)-inducible promoter (Figure 3e).

With delivery of bumped substrates and BH-GalNAc-T expression as crucial prerequisites, the stage was set to test incorporation of GalNAc analogs into cell surface glycans. Using the alkyne group as a bioorthogonal tag is not without caveats, as it relies on using CuAAC instead of SPAAC for reaction with reporter moieties such as fluorophores [6]. Wu and coworkers had developed biocompatible Cu(I) ligands to allow for CuAAC on the surface of living cells 45, 46. Picolyl azide derivatives developed by Ting and colleagues [47] that accelerate CuAAC by an order of magnitude served to characterize cell surface glycans introduced by individual GalNAc-Ts by both flow cytometry and in-gel fluorescence (Figure 3e). We found a several-fold increase of signal over background when a functional BH system was present, and a largely overlapping band pattern of labeled glycoproteins between GalNAc-T1 and T2. MS proteomics revealed that these were glycoproteins with highly O-glycosylated mucin domains, rendering them potential substrates of both isoenzymes T1 and T2 [11]. However, certain reproducible differences between T1- and T2-labeled band patterns indicated that substrates are specifically modified by individual isoenzymes.

Of note, the dependence on both AGX1^F383A^ and BH-GalNAc-Ts for efficient labeling allowed us to assess background labeling in the absence of either enzyme. We could thus rule out nonspecific fluorescence signal associated with an elimination–addition reaction on certain MOE reagents and confirm the validity of our strategy (Figure 3e) [48]. In contrast, we observed a notable background when bumped UDP-GalNAc analog **3** was biosynthesized but BH-GalNAc-Ts were not present. Nucleotide-sugar profiling revealed that a small portion of **3** is epimerized to the corresponding UDP-GlcNAc analog by GALE. Hypothesizing that N-linked glycans are the main destination of GlcNAc analogs on the cell surface, we substantially reduced background labeling by treating cell lysates with the de-N-glycanase PNGase F. Fluorescent labeling signal in the absence of Dox-induced BH-GalNAc-T expression resembled background signal, confirming the suitability of GalNAc-T BH engineering in K-562 cells (Figure 3e). We have since found that the use of branched acylamide side chains renders UDP-GalNAc derivatives resistant toward epimerization (Figure 2a), resulting in the probe UDP-GalNAzMe **6** (Figure 2c) that is specific for O-GalNAc glycans [12].

Creating gain-of-function reporter tools for the activity of individual GalNAc-Ts, the BH tactic provides a unique opportunity to directly analyze GalNAc-T-specific glycosylation sites when integrated into state-of-the art MS glycoproteomics workflows.

### MS glycoproteomics

The field of MS glycoproteomics has seen a multitude of recent technical and conceptual advances [49, 50, 51, 52, 53, 54, 55, 56]. However, the primary focus to map glycosylation sites still faces many challenges, especially when compared to traditional peptide-centered proteomics. One of the biggest challenges is the structural complexity of the glycome [57]. Glycans are made up of relatively few monosaccharide units, but the large number of possible branching sites, combined with the possible linkage stereochemistry, creates an enormous number of possible structures [58]. Further, these glycans can be found on several possible residues throughout the protein. In general, the complexity of glycans is compounded by issues with glycoprotein enrichment, instrumentation, and data analysis [59,60].

### Enrichment

**Figure 4:**
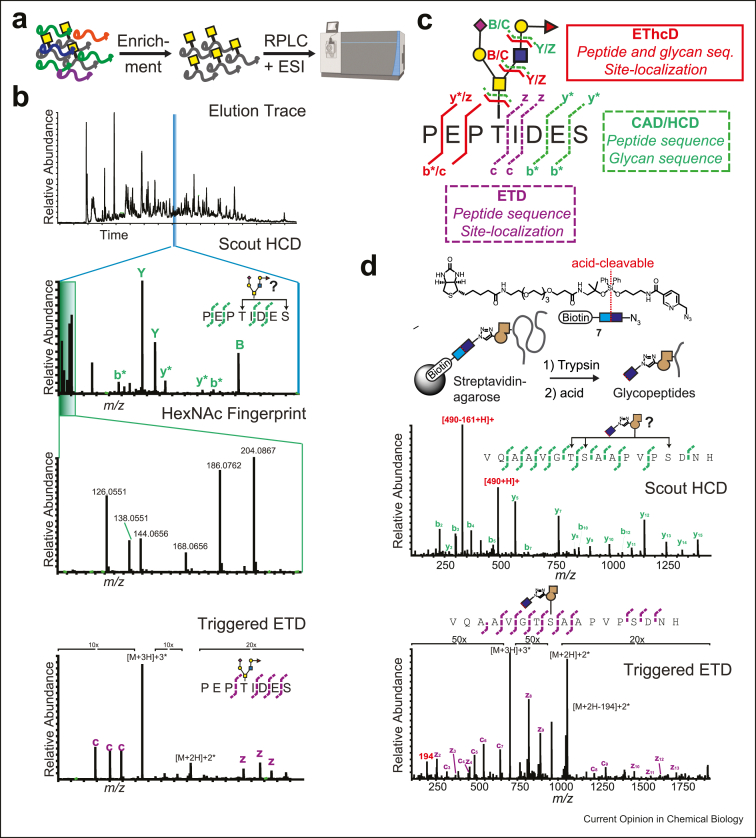
**Glycoproteomic workflows enable analysis of the glycoproteome complexity. (a)** Typical workflow before MS analysis. The complex sample contains unmodified peptides, differentially modified peptides, and glycopeptides. To enrich for the latter, samples are subjected to lectin columns, SPE extraction, or chemical enrichment procedures. The elution is then separated by RP-HPLC and electrospray ionized into the mass spectrometer. (**b)** Schematic of the instrumentation parameters often used in glycoproteomic analysis. The RP-HPLC elution trace is shown in the top panel and consists of a series of full mass spectra (MS1s). Typically, ions are selected in an abundance-dependent manner and subjected to HCD fragmentation (2nd panel). If a glycopeptide is present, a HexNAc fingerprint will be visible (3rd panel), which can then be used to trigger electron-based fragmentation (bottom panel). (**c)** Overview of glycopeptide fragmentation methods and the information they can provide. ETD (purple dashes) only fragments the peptide backbone, giving complementary c/z type ions with the glycan attached to the peptide. HCD (green dashes) fragments the peptide backbone as well as the glycosidic linkages, allowing for peptide and glycan sequencing, but often loses site-specificity. EThcD (red lines) combines the two techniques and allows for the most information to be gleaned from a single spectrum. Adapted from a study by Reiding et al. [92]. (**d)** Bump-and-hole chemical glycoproteomics provides a gain-of-function enrichment strategy (top). An HCD spectrum is shown under this, where 2 new fingerprint ions are present (491, 330) that can be used to trigger ETD (bottom). The ETD spectrum allows for site-localization of the modification and also demonstrates a new fingerprint ion (194) that can be used in search algorithms for more confident scoring. SPE, solid-phase extraction; RP, reverse-phase; HCD, higher-energy collisional dissociation; ETD, Electron transfer dissociation; HPLC, high performance liquid chromatography.

Glycoproteins are typically present at low abundance in complex samples, necessitating an enrichment step prior to MS analysis (Figure 4a) [61]. Types of enrichment broadly fall into three categories: affinity-based, solid-phase extraction (SPE), and chemical methods. Affinity-based methods are most commonly used and involve lectins and/or antibodies to enrich glycoconjugates. Lectins are glycan-binding proteins that enrich for particular glycan structures and have found use in numerous studies [62, 63, 64, 65] but can be limited by low binding affinity and poor specificity. Generally, lectins are not well-suited for untargeted glycoproteomics unless used in combination or succession.

To overcome these issues, SPE techniques have been developed, such as hydrophilic interaction chromatography (HILIC). Here, a hydrophilic stationary phase with a hydrophobic mobile phase preferentially retains hydrophilic glycopeptides [66, 67, 68]. When compared with lectins, HILIC has a much higher glycan promiscuity, enabling enrichment of a wide array of glycopeptides. In addition, several groups have investigated zwitterionic (ZIC-HILIC), [69, 70, 71], electrostatic repulsion liquid interaction chromatography (ERLIC) [72,73], and strong anion exchange (SAX-ERLIC) with HILIC to increase enrichment effectiveness and coverage of the glycoproteome. Other types of SPE include boronic acids that form covalent bonds with vicinal *cis*-diols on glycans [74, 75, 76, 77], and titanium dioxide resins that are specific for negatively charged glycans [78, 79, 80].

Chemical enrichment methods involve derivatization or metabolic labeling of glycans, often followed by tagging the moiety with a secondary reporter, and then enriching for the glycopeptides [81]. One of the first examples was introduced by Aebersold et al. [82, 83, 84, 85], where sialic acids were periodate-oxidized and enriched using hydrazide-functionalized beads. One drawback of this procedure is loss of glycan structural information. Enrichment of bioorthogonal MOE reagents is discussed below and in specialized reviews [6].

### Fragmentation

Enriched glycopeptides are usually subjected to liquid chromatography (LC) followed by tandem MS analysis (Figure 4a). Various LC technologies have been reviewed elsewhere [86.∗∗, 87, 88, 89]. The most common workflows use reverse-phase high performance liquid chromatography (HPLC) coupled to electrospray ionization. Glycopeptides are then subjected to different types of fragmentation (i.e., tandem MS) to sequence the peptide, identify the glycan, and site-localize the glycosylation position. Collision-activated dissociation occurs when a peptide is subjected to collisions with helium atoms [90]. Beam-type collision-activated dissociation, called higher-energy collisional dissociation (HCD) on Orbitrap instruments, is similar but uses nitrogen instead of helium [91]. Each one of these collisions imparts vibrational energy to the peptide, eventually leading to cleavage of the most labile bond in the peptide. For glycopeptides, the most labile bonds are the glycosidic bonds (i) between peptide and glycan, and/or (ii) between individual monosaccharides (Figure 4c). Thus, the spectra generated are dominated by the losses of glycans from the peptide, protonated glycan species, and/or naked b-type and y-type peptide ions [86,92,93].

Electron transfer dissociation (ETD) was developed, in part, to overcome the issue of predominant glycan fragmentation and allow for unambiguous glycosylation site localization [94,95]. Because this reaction does not rely on collision for fragmentation, the glycan remains attached to the peptide backbone and allows for site-localization of glycosylation (Figure 4c) [31,96]. As a downside, ETD relies on high charge density, which often is not the case in large (i.e. >1000 *m/z*) glycopeptide precursor ions. To overcome this challenge, several groups have implemented supplemental activation in ETD, including EThcD [92,97,98] and activated ion ETD (AI-ETD) [63,99].

The two most commonly used fragmentation techniques in glycoproteomics are (i) stepped-collision energy HCD, and (ii) HCD-triggered ETD or EThcD (HCD-pd-ETD) [93]. The former is most common and recommended for N-glycoproteomics, whereas the latter is necessary for O-glycoproteomics. In stepped-collision energy HCD, three collision energies are used on the same precursor and product ions are accumulated and analyzed together [100,101]. In HCD-pd-ETD, HCD is first performed on all precursor ions. HexNAc (GlcNAc or GalNAc) ions will generate a fingerprint consisting of 6 masses that can then be used to trigger ETD or EThcD, which often allows for unambiguous site localization of the glycan (Figure 4b) [102,103]. Chemical glycan modifications contribute additional trigger ions that can be beneficial (*see* in the following context).

### Data analysis

A fundamental challenge of glycoproteomics is data analysis. Often, spectra are input into a search algorithm to assist data interpretation. While most search engines were developed for the identification of unmodified peptides and struggle with glycopeptides [104], some take glycosylation into account. Byonic is the current glycoproteomic ‘gold standard’ [105], although some reports suggest that Protein Prospector may be better at site-localization of O-glycans [106]. Recently, ‘O-Pair Search’ and ‘MSFragger’ programs have been introduced and display great promise in confident localization and scoring of glycoproteomic spectra [107,108]. Several thorough reviews have assembled available glycobioinformatic tools [109, 110∗, 111]. Importantly, at this point, expert manual interpretation is still required to confidently assign glycan composition and site of attachment [60]. Glycoproteomics is in need of new search algorithms and databases to overcome this time consuming and low-throughput step. Chemical tools could be fundamental to this end, for instance, through introducing isotope-based glycoproteomics workflows [29,112,113].

### Other developments in the field of glycoproteomics

We envision that several techniques currently in development will have a strong impact on glycoproteomics. For instance, advances in glycopeptide ionization are sorely needed to better analyze glycoconjugates [114, 115, 116∗]. Furthermore, at the moment, glycoproteomics cannot inherently ascertain glycan stereochemistry and linkage. New methods, such as ultraviolet photodissociation [117,118], infrared spectroscopy–MS and/or infrared multiphoton dissociation [119,120], or data independent acquisition [121,122] might allow for this information to be ascertained in a single glycoproteomics run. Furthermore, ion-mobility MS helps separate isomeric structures, which can lead to less convoluted glycopeptide spectra [58,123,124]. Finally, to catapult glycoproteomics to new heights, we need more straightforward methods to validate MS results in order to allow for facile investigation into the biological relevance of glycosylation [125].

### The promise of chemical precision tools to simplify MS glycoproteomics

Ever since their advent in the late 1990s, MOE reagents have allowed for an alternative view into the glycome [135]. Bioorthogonal groups are ideal for modern chemical proteomics techniques [6]. Most approaches rely on biotin-containing clickable handles for glycopeptide enrichment before MS glycoproteomics. To specifically enrich glycopeptides, a neutravidin-based on-resin proteolytic digestion protocol is often used to remove any nonglycosylated peptides. The biotin-picolyl azide reagent **7** has an additional acid-cleavable diphenyldisiloxane group developed by Tirrell et al. [112,113,126,127], and since then has been applied to glycoproteomics (Figure 4d). In our hands, the bioorthogonal handle offered advantages during data acquisition as well as validation, as new fingerprint ions appeared during HCD and were used to trigger ETD. In the ETD spectrum, another fingerprint ion (194 *m/z*) was present, which served to validate spectra and could be used in search algorithms for more confident glycan assignment [11].

The advantages of chemical glycoproteomics fueled by the gain-of-function nature of BH engineering enabled us to directly map the glycosylation sites primed by two GalNAc-T isoenzymes. We used HepG2 cells engineered to lack native expression of T1 or T2 and complemented with BH-T1 and -T2, respectively [128]. Upon biosynthesis of UDP-GalNAc analog **3**, differential glycosylation sites primed by both isoenzymes were mapped. Among these, several had been suggested to be isoenzyme-specific by Schjoldager et al. [128] using SimpleCells and thereby benchmarked our approach, while others were new. The added advantage of the BH tactic showed in two pieces of data that had been previously impossible to accrue: (i) our data resolved ambiguity about isoenzyme specificity of apolipoprotein AI glycosylation, which had been impaired by events of compensation and competition within the GalNAc-T family; and (ii) the chemically modified GalNAc analog was elaborated up to the tetrasaccharide (Neu5Ac)_2_-Hex-GalNAc, in accordance with the so-called di-Sialyl-T antigen as the largest abundant O-GalNAc glycan made in HepG2 cells [129]. As the enrichment process based on biotinylation should be unbiased to glycan substructures, mapping glycan elaboration offers exciting opportunities to potentially combine site annotation and glycome analysis, which is currently challenging on a glycoproteome-wide scale.

### Outlook

The success of the BH tactic suggests important implications for studying one of the most fascinating and obscure GT families. As the tactic relies on mutating gatekeeper residues that are conserved throughout the family, in principle, it should be applicable to other isoenzymes. Multiple GalNAc-Ts have been crystallized over the last years [43,130∗, 131∗, 132∗, 133∗], and available structures seem to confirm this notion. The field of chemical glycoproteomics is rapidly evolving, constantly increasing the sensitivity of MS analysis. Current challenges associated with optimizing glycopeptide enrichment, purification, and ionizability will be tackled within the next few years to truly deliver the promise of chemical precision tools to quantitative biology [134].

## Declaration of competing interest

The authors declare that they have no known competing financial interests or personal relationships that could have appeared to influence the work reported in this paper.

## References

[1] Varki A., Gagneux P., Varki A., Cummings R.D., Esko J.D. (2017). Biological functions of glycans. Essentials of glycobiology.

[2] Raman R., Raguram S., Venkataraman G., Paulson J.C., Sasisekharan R. (2005). Glycomics: an integrated systems approach to structure-function relationships of glycans. Nat Methods.

[3] Taylor M.E., Drickamer K. (2011). Introduction to glycobiology.

[4] Jaiman A., Thattai M. (2020). Golgi compartments enable controlled biomolecular assembly using promiscuous enzymes. eLife.

[5] Zilmer M., Edmondson A.C., Khetarpal S.A., Alesi V., Zaki M.S., Rostasy K., Madsen C.G., Lepri F.R., Sinibaldi L., Cusmai R. (2020). Novel congenital disorder of O-linked glycosylation caused by GALNT2 loss of function. Brain.

[bib6] Parker C.G., Pratt M.R. (2020). Primer click chemistry in proteomic investigations. Cell.

[7] Hanson S.R., Hsu T.-L., Weerapana E., Kishikawa K., Simon G.M., Cravatt B.F., Wong C.-H. (2007). Tailored glycoproteomics and glycan site mapping using saccharide-selective bioorthogonal probes. J Am Chem Soc.

[8] Saxon E., Bertozzi C.R. (2007). Cell surface engineering by a modified staudinger reaction. Science.

[9] Laughlin S.T., Bertozzi C.R. (2009). Imaging the glycome. Proc Natl Acad Sci.

[10] Patterson D.M., Nazarova L.A., Xie B., Kamber D.N., Prescher J.A. (2012). Functionalized cyclopropenes as bioorthogonal chemical reporters. J Am Chem Soc.

[bib11] Schumann B., Malaker S.A., Wisnovsky S.P., Debets M.F., Agbay A.J., Fernandez D., Wagner L.J.S., Lin L., Li Z., Choi J. (2020). Bump-and-Hole engineering identifies specific substrates of glycosyltransferases in living cells. Mol Cell.

[bib12] Debets M.F., Tastan O.Y., Wisnovsky S.P., Malaker S.A., Angelis N., Moeckl L.K.R., Choi J., Flynn H., Wagner L.J.S., Bineva-Todd G. (2020). Metabolic precision labeling enables selective probing of O-linked N-acetylgalactosamine glycosylation. Proc Natl Acad Sci USA.

[13] Möckl L., Pedram K., Roy A.R., Krishnan V., Gustavsson A.K., Dorigo O., Bertozzi C.R., Moerner W.E. (2019). Quantitative super-resolution microscopy of the mammalian glycocalyx. Dev Cell.

[bib14] Boyce M., Carrico I.S., Ganguli A.S., Yu S.H., Hangauer M.J., Hubbard S.C., Kohler J.J., Bertozzi C.R. (2011). Metabolic cross-talk allows labeling of O-linked β-N- acetylglucosamine-modified proteins via the N-acetylgalactosamine salvage pathway. Proc Natl Acad Sci.

[15] Alaimo P.J., Shogren-knaak M.A., Shokat K.M. (2001). Chemical genetic approaches for the elucidation of signaling pathways. Curr Opin Chem Biol.

[16] Hertz N.T., Wang B.T., Allen J.J., Zhang C., Dar A.C., Burlingame A.L., Shokat K.M. (2014). Chemical genetic approach for kinase-substrate mapping by covalent capture of thiophosphopeptides and analysis by mass spectrometry. Curr Protoc Chem Biol.

[17] Yang C., Mi J., Feng Y., Ngo L., Gao T., Yan L., Zheng Y.G. (2014). Labeling lysine acetyltransferase substrates with engineered enzymes and functionalized cofactor surrogates. J Am Chem Soc.

[18] Blum G., Bothwell I.R., Islam K., Luo M. (2015). Profiling protein methylation with cofactor Analogue containing terminal alkyne functionality. Curr Protoc Chem Biol.

[19] Carter-O’Connell I., Jin H., Morgan R.K., David L.L., Cohen M.S. (2017). Engineering the substrate specificity of ADP-ribosyltransferases for identifying direct protein targets. Physiol Behav.

[20] Wang L., Brock A., Herberich B., Schultz P.G. (2001). Expanding the genetic code of Escherichia coli. Science.

[21] Luther K.B., Schindelin H., Haltiwanger R.S. (2009). Structural and mechanistic insights into lunatic fringe from a kinetic analysis of enzyme mutants. J Biol Chem.

[22] Seto N.O.L., Palcic M.M., Compston C.A., Li H., Bundle D.R., Narang S.A. (1997). Sequential interchange of four amino acids from blood group B to blood group A glycosyltransferase boosts catalytic activity and progressively modifies substrate recognition in human recombinant enzymes. J Biol Chem.

[23] Tumbale P., Jamaluddin H., Thiyagarajan N., Acharya K.R., Brew K. (2008). Screening a limited structure-based library identifies UDP-GalNAc-specific mutants of α-1,3-galactosyltransferase. Glycobiology.

[24] Marcus S.L., Polakowski R., Seto N.O.L., Leinala E., Borisova S., Blancher A., Roubinet F., Evans S.V., Palcic M.M. (2003). A single point mutation reverses the donor specificity of human blood group B-synthesizing galactosyltransferase. J Biol Chem.

[bib25] Ramakrishnan B., Qasba P.K. (2002). Structure-based design of β1,4-galactosyltransferase I (β4Gal-T1) with equally efficient N-acetylgalactosaminyltransferase activity: point mutation broadens β4Gal-T1 donor specificity. J Biol Chem.

[26] Boeggeman E., Ramakrishnan B., Kilgore C., Khidekel N., Hsieh-Wilson L.C., Simpson J.T., Qasba P.K. (2007). Direct identification of nonreducing GlcNAc residues on N-glycans of glycoproteins using a novel chemoenzymatic method. Bioconjug Chem.

[27] Qasba P.K., Boeggeman E., Ramakrishnan B. (2008). Site-specific linking of biomolecules via glycan residues using glycosyltransferases. Biotechnol Prog.

[28] Vocadlo D.J., Hang H.C., Kim E., Hanover J.A., Bertozzi C.R. (2003). A chemical approach for identifying O-GlcNAc- modified proteins in cells. Proc Natl Acad Sci.

[29] Woo C.M., Iavarone A.T., Spiciarich D.R., Palaniappan K.K., Bertozzi C.R. (2015). Isotope-targeted glycoproteomics (IsoTaG ): a mass-independent platform for intact N- and O-glycopeptide discovery and analysis. Nat Methods.

[30] Alfaro J.F., Gong C.X., Monroe M.E., Aldrich J.T., Clauss T.R.W., Purvine S.O., Wang Z., Camp D.G., Shabanowitz J., Stanley P. (2012). Tandem mass spectrometry identifies many mouse brain O-GlcNAcylated proteins including EGF domain-specific O-GlcNAc transferase targets. Proc Natl Acad Sci.

[31] Wang Z., Udeshi N.D., Malley M.O., Shabanowitz J., Hunt D.F., Hart G.W. (2010). Enrichment and site mapping of O-linked N-acetylglucosamine by a combination of chemical/enzymatic tagging, photochemical cleavage, and electron transfer dissociation mass spectrometry. Mol Cell Proteomics.

[32] Burnham-Marusich A.R., Snodgrass C.J., Johnson A.M., Kiyoshi C.M., Buzby S.E., Gruner M.R., Berninsone P.M. (2012). Metabolic labeling of Caenorhabditis elegans primary embryonic cells with azido-sugars as a tool for glycoprotein discovery. PloS One.

[bib33] Khidekel N., Arndt S., Lamarre-vincent N., Lippert A., Poulin-kerstien K.G., Ramakrishnan B., Qasba P.K., Hsieh-wilson L.C. (2003). A chemoenzymatic approach toward the rapid and sensitive detection of O-GlcNAc posttranslational modifications. J Am Chem Soc.

[34] Thompson J.W., Griffin M.E., Hsieh-Wilson L.C. (2019). Methods for the detection, study, and dynamic profiling of O-GlcNAc glycosylation. Methods Enzymol.

[35] Darabedian N., Thompson J.W., Chuh K.N., Hsieh-Wilson L.C., Pratt M.R. (2018). Optimization of chemoenzymatic mass tagging by strain-promoted cycloaddition (SPAAC) for the determination of O-GlcNAc stoichiometry by western blotting. Biochemistry.

[bib36] Pouilly S., Bourgeaux V., Piller F. (2012). Evaluation of analogues of GalNAc as substrates for enzymes of the mammalian GalNAc salvage pathway. ACS Chem Biol.

[bib37] Yu S., Boyce M., Wands A.M., Bond M.R., Bertozzi C.R., Kohler J.J. (2012). Metabolic labeling enables selective photocrosslinking of O-GlcNAc-modified proteins to their binding partners. Proc Natl Acad Sci.

[38] Rodriguez A.C., Yu S., Li B., Zegzouti H., Kohler J.J., Corporation P. (2015). Enhanced transfer of a photocrosslinking GlcNAc analog by an O-GlcNAc transferase mutant with converted substrate specificity. J Biol Chem.

[39] Kingsley P.D., Ten Hagen K.G., Maltby K.M., Zara J., Tabak L.A. (2000). Diverse spatial expression patterns of UDP-GalNAc: polypeptide N-acetylgalactosaminyl-transferase family member mRNAs during mouse development. Glycobiology.

[40] Peng R.Q., Wan H.Y., Li H.F., Liu M., Li X., Tang H. (2012). MicroRNA-214 suppresses growth and invasiveness of cervical cancer cells by targeting UDP-N-acetyl-α-D-galactosamine:polypeptide N- acetylgalactosaminyltransferase 7. J Biol Chem.

[41] Lavrsen K., Dabelsteen S., Vakhrushev S.Y., Levann A.M.R., Haue A.D., Dylander A., Mandel U., Hansen L., Frodin M., Bennett E.P. (2018). De novo expression of human polypeptide N-acetylgalactosaminyltransferase 6 (GalNAc-T6) in colon adenocarcinoma inhibits the differentiation of colonic epithelium. J Biol Chem.

[42] Khetarpal S.A., Schjoldager K.T., Christoffersen C., Edmondson A.C., Reutter H.M., Ahmed B., Peloso G.M., Vitali C., Zhao W., Hanasoge A.V. (2017). Loss of function of GALNT2 lowers high density lipoproteins in humans, nonhuman primates, and rodents. Cell Metab.

[bib43] Kubota T., Shiba T., Sugioka S., Furukawa S., Sawaki H., Kato R., Wakatsuki S., Narimatsu H. (2006). Structural basis of carbohydrate transfer activity by human UDP-GalNAc: polypeptide α-N-acetylgalactosaminyltransferase (pp-GalNAc-T10). J Mol Biol.

[bib44] Choi J., Wagner L.J.S., Timmermans S.B.P.E., Malaker S.A., Schumann B., Gray M.A., Debets M.F., Takashima M., Gehring J., Bertozzi C.R. (2019). Engineering orthogonal polypeptide GalNAc-transferase and UDP- sugar pairs. J Am Soc Chem.

[45] Wang W., Hong S., Tran A., Jiang H., Triano R., Yan A., Liu Y., Chen X., Wu P. (2011). Sulfated ligands for the copper(I)-catalyzed azide-alkyne cycloaddition. Chem Asian J.

[46] Besanceney-Webler C., Jiang H., Zheng T., Feng L., Soriano del Amo D., Wang W., Klivansky L.M., Marlow F.L., Liu Y., Wu P. (2011). Increasing the efficacy of bioorthogonal click reactions for bioconjugation: a comparative study. Angew Chem Int Ed.

[47] Uttamapinant C., Tangpeerachaikul A., Grecian S., Clarke S., Singh U., Slade P., Gee K.R., Ting A.Y. (2012). Fast, cell-compatible click chemistry with copper-chelating azides for biomolecular labeling. Angew Chem.

[48] Qin K., Zhang H., Zhao Z., Chen X. (2020). Protein S-Glyco-Modification through an elimination-addition mechanism protein S-Glyco-Modification through an elimination-addition mechanism. J Am Chem Soc.

[49] Trinidad J.C., Schoepfer R., Burlingame A.L., Medzihradszky K.F. (2013). N- and O-Glycosylation in the murine synaptosome. Mol Cell Proteomics.

[50] Shah P., Wang X., Yang W., Eshghi S.T., Sun S., Hoti N., Chen L., Yang S., Pasay J., Rubin A. (2015). Integrated proteomic and glycoproteomic analyses of prostate cancer cells reveal glycoprotein alteration in protein abundance and glycosylation. Mol Cell Proteomics.

[51] Medzihradszky K.F., Kaasik K., Chalkley R.J. (2015). Tissue-specific glycosylation at the glycopeptide level. Mol Cell Proteomics.

[52] Yu A., Zhao J., Peng W., Banazadeh A., Williamson S.D., Goli M., Huang Yifan, Mechref Y. (2018). Advances in mass spectrometry-based glycoproteomics. Electrophoresis.

[53] Rudd P., Karlsson N.G., KhooKH, Packer N.H., Varki A., Cummings R.D., Esko J.D. (2017). Glycomics and glycoproteomics. Essentials of glycobiology.

[54] Shajahan A., Heiss C., Ishihara M., Azadi P. (2017). Glycomic and glycoproteomic analysis of glycoproteins-a tutorial. Anal Bioanal Chem.

[55] Alagesan K., Everest-Dass A., Kolarich D. (2018). Isomeric separation and characterisation of glycoconjugates. Adv Exp Med Biol.

[56] Li Q., Xie Y., Wong M., Lebrilla C. (2019). Characterization of cell glycocalyx with mass spectrometry methods. Cells.

[bib57] Khoo K.-H. (2019). Advances toward mapping the full extent of protein site-specific O-GalNAc glycosylation that better reflects underlying glycomic complexity. Curr Opin Struct Biol.

[58] Mookherjee A., Guttman M. (2018). Bridging the structural gap of glycoproteomics with ion mobility spectrometry. Curr Opin Chem Biol.

[59] Chen Z., Huang J., Li L. (2019). Recent advances in mass spectrometry (MS)-based glycoproteomics in complex biological samples. Trends Anal Chem.

[bib60] Thaysen-Andersen M., Packer N.H., Schulz B.L. (2016). Maturing glycoproteomics technologies provide unique structural insights into the N -glycoproteome and its regulation in health and disease. Mol Cell Proteomics.

[61] Chandler K.B., Costello C.E. (2016). Glycomics and glycoproteomics of membrane proteins and cell-surface receptors: present trends and future opportunities. Electrophoresis.

[62] Steentoft C., Vakhrushev S.Y., Joshi H.J., Kong Y., Vester-Christensen M.B., Schjoldager K.T.-B.G., Lavrsen K., Dabelsteen S., Pedersen N.B., Marcos-SilvaL, Gupta R., Bennet E.P., Mandel U., Brunak S., Wandall H.H., Levery S.B., Clausen H. (2013). Precision mapping of the human O-GalNAc glycoproteome through SimpleCell technology. EMBO J.

[63] Riley N.M., Hebert A.S., Westphall M.S., Coon J.J. (2019). Capturing site-specific heterogeneity with large-scale N-glycoproteome analysis. Nat Commun.

[64] Xiao H., Chen W., Smeekens J.M., Wu R. (2018). An enrichment method based on synergistic and reversible covalent interactions for large-scale analysis of glycoproteins. Nat Commun.

[65] Trinidad J.C., Barkan D.T., Gulledge B.F., Thalhammer A., Sali A., Schoepfer R., Burlingame A.L. (2012). Global identification and characterization of both O -GlcNAcylation and phosphorylation at the murine synapse. Mol Cell Proteomics.

[66] Sun N., Wu H., Chen H., Shen X., Deng C. (2019). Advances in hydrophilic nanomaterials for glycoproteomics. Chem Commun.

[67] Jensen P.H., Mysling S., Højrup P., Jensen O.N. (2013). Glycopeptide enrichment for MALDI-TOF mass spectrometry analysis by hydrophilic interaction liquid chromatography solid phase extraction (HILIC SPE). Methods Mol Biol.

[68] Scott N.E., Parker B.L., Connolly A.M., Paulech J., Edwards A.V.G., Crossett B., Falconer L., Kolarich D., Djordjevic S.P., Højrup P., Packer N.H., Larsen M.R., Cordwell S.J. (2011). Simultaneous glycan-peptide characterization using hydrophilic interaction chromatography and parallel fragmentation by CID, higher energy collisional dissociation, and electron transfer dissociation MS applied to the N -linked glycoproteome of campylobac. Mol Cell Proteomics.

[69] Calvano C.D., Zambonin C.G., Jensen O.N. (2008). Assessment of lectin and HILIC based enrichment protocols for characterization of serum glycoproteins by mass spectrometry. J Proteomics.

[70] CaoW, Huang J., Jiang B., Gao X., Yang P. (2016). Highly selective enrichment of glycopeptides based on zwitterionically functionalized soluble nanopolymers. Sci Rep.

[71] Alagesan K., Khilji S.K., Kolarich D. (2017). It is all about the solvent: on the importance of the mobile phase for ZIC-HILIC glycopeptide enrichment. Anal Bioanal Chem.

[72] Sok Hwee Cheow E., Sim K.H., de Kleijn D., Lee C.N., Sorokin V., Sze S.K. (2015). Simultaneous enrichment of plasma soluble and extracellular vesicular glycoproteins using prolonged ultracentrifugation-electrostatic repulsion-hydrophilic interaction chromatography (PUC-ERLIC) approach. Mol Cell Proteomics.

[73] Alpert A.J. (2008). Electrostatic repulsion hydrophilic interaction chromatography for isocratic separation of charged solutes and selective isolation of phosphopeptides. Anal Chem.

[74] Chen W., Smeekens J.M., Wu R. (2014). A universal chemical enrichment method for mapping the yeast N -glycoproteome by mass spectrometry (MS). Mol Cell Proteomics.

[75] Sparbier K., Wenzel T., Kostrzewa M. (2006). Exploring the binding profiles of ConA, boronic acid and WGA by MALDI-TOF/TOF MS and magnetic particles. J Chromatogr B Analyt Technol Biomed Life Sci.

[76] Zhang Q., Tang N., Brock J.W.C., Mottaz H.M., Ames J.M., Baynes J.W., Smith R.D., Metz T.O. (2007). Enrichment and analysis of nonenzymatically glycated peptides: boronate affinity chromatography coupled with electron-transfer dissociation mass spectrometry. J Proteome Res.

[77] Xu Y., Wu Z., Zhang L., Lu H., Yang P., Webley P.A., Zhao D. (2009). Highly specific enrichment of glycopeptides using boronic acid-functionalized mesoporous silica. Anal Chem.

[78] Palmisano G., Eun Lendal S., Engholm-Keller K., Leth-Larsen R., Parker B.L., Larsen M.R. (2010). Selective enrichment of sialic acid–containing glycopeptides using titanium dioxide chromatography with analysis by HILIC and mass spectrometry. Nat Protoc.

[79] Larsen M.R., Jensen S.S., Jakobsen L.A., Heegaard N.H.H. (2007). Exploring the sialiome using titanium dioxide chromatography and mass spectrometry. Mol Cell Proteomics.

[80] Yan J., Li X., Yu L., Kin Y., Zhang X., Xue X., Ke Y., Liang X. (2010). Selective enrichment of glycopeptides/phosphopeptides using porous titania microspheres. Chem Commun.

[bib81] Palaniappan K.K., Bertozzi C.R. (2016). Chemical glycoproteomics. Chem Rev.

[82] Zhang H., Li X., Martin D.B., Aebersold R. (2003). Identification and quantification of N-linked glycoproteins using hydrazide chemistry, stable isotope labeling and mass spectrometry. Nat Biotechnol.

[83] Nilsson J., Rüetschi U., Halim A., Hesse C., Carlsohn E., Brinkmalm, Larson G. (2009). Enrichment of glycopeptides for glycan structure and attachment site identification. Nat Methods.

[84] Halim A., Rüetschi U., Larson G., Nilsson J. (2013). LC–MS/MS characterization of O-glycosylation sites and glycan structures of human cerebrospinal fluid glycoproteins. J Proteome Res.

[85] Kurogochi M., Matsushista T., Amano M., Furukawa J., Shinohara Y., Aoshima M., Nishimura S.-I. (2010). Sialic acid-focused quantitative mouse serum glycoproteomics by multiple reaction monitoring assay. Mol Cell Proteomics.

[bib86] Ruhaak L.R., Xu G., Li Q., Goonatilleke E., Lebrilla C.B. (2018). Mass spectrometry approaches to glycomic and glycoproteomic analyses. Chem Rev.

[87] Alagesan K., Everest-Dass A., Kolarich D., Yamaguchi Y., Kato K. (2018). Isomeric separation and characterisation of glycoconjugates. Glycobiophysics.

[88] Camperi J., Pichon V., Delaunay N. (2020). Separation methods hyphenated to mass spectrometry for the characterization of the protein glycosylation at the intact level. J Pharm Biomed Anal.

[89] Huang B.-Y., Yang C.-K., Liu C.-P., Liu C.-Y. (2014). Stationary phases for the enrichment of glycoproteins and glycopeptides: liquid Phase Separations. Electrophoresis.

[90] Wells J.M., McLuckey S.A. (2005). Collision-induced dissociation (CID) of peptides and proteins. Methods Enzymol.

[91] Olsen J.V., Macek B., Lange O., Makarov A., Horning S., Mann M. (2007). Higher-energy C-trap dissociation for peptide modification analysis. Nat Methods.

[bib92] Reiding K.R., Bondt A., Franc V., Heck A.J.R. (2018). The benefits of hybrid fragmentation methods for glycoproteomics. Trends Anal Chem.

[bib93] Riley N.M., Malaker S.A., Driessen M., Bertozzi C.R. (2020). Optimal dissociation methods differ for N- and O-glycopeptides. J Proteome Res.

[94] Coon J.J., Syka J.E.P., Shabanowitz J., Hunt D.F. (2005). Tandem mass spectrometry for peptide and protein sequence analysis. Biotechniques.

[95] Syka J.E.P., Coon J.J., Schroeder M.J., Shabanowitz J., Hunt D.F. (2004). Peptide and protein sequence analysis by electron transfer dissociation mass spectrometry. Proc Natl Acad Sci.

[96] Myers S.A., Daou S., Affar E.B., Burlingame A. (2013). Electron transfer dissociation (ETD): the mass spectrometric breakthrough essential for O -GlcNAc protein site assignments-a study of the O -GlcNAcylated protein Host Cell Factor C1. Proteomics.

[97] Yang Y., Franc V., Heck A.J.R. (2017). Glycoproteomics: a balance between high-throughput and in-depth analysis. Trends Biotechnol.

[98] Yu Q., Canales A., Glover M.S., Das R., Shi X., Liu Y., Keller M.P., Attie A.D., Li Lingiun (2017). Targeted mass spectrometry approach enabled discovery of O- glycosylated insulin and related signaling peptides in mouse and human pancreatic islets. Anal Chem.

[99] Riley N.M., Westphall M.S., Coon J.J. (2017). Activated ion-electron transfer dissociation enables comprehensive top-down protein fragmentation. J Proteome Res.

[100] Cao L., Tolić N., Qu Y., Mend D., Zhao R., Zhang Q., Moore R.J., Zink E.M., Lipton M.S., Paša-Tolić L., Wu S. (2014). Characterization of intact N- and O-linked glycopeptides using higher energy collisional dissociation. Anal Biochem.

[101] Hinneburg H., Stavenhagen K., Schweiger-Hufnagel U., Pengelley S., Jabs W., Seeberger P.H., Varón Silva D., Wuhrer M., Kolarich D. (2016). The art of destruction: optimizing collision energies in quadrupole-time of flight (Q-TOF) instruments for glycopeptide-based glycoproteomics. J Am Soc Mass Spectrom.

[102] Wu S.-W., Pu T.-H., Viner R., Khoo K.-H. (2014). Novel LC-MS2 product dependent parallel data acquisition function and data analysis workflow for sequencing and identification of intact glycopeptides. Anal Chem.

[103] Singh C., Zampronio C.G., Creese A.J., Cooper H.J. (2012). Higher energy collision dissociation (HCD) product ion-triggered electron transfer dissociation (ETD) mass spectrometry for the analysis of N -linked glycoproteins. J Proteome Res.

[104] Darula Z., Medzihradszky K.F. (2018). Analysis of mammalian O-Glycopeptides-We have made a good start, but there is a long way to go. Mol Cell Proteomics.

[105] Bern M., Kil Y.J., Becker C. (2012). Byonic: advanced peptide and protein identification software. Curr Protoc Bioinformatics.

[106] Pap A., Klement E., Hunyadi-Gulyas E., Darula Z., Medzihradszky K.F. (2018). Status report on the high-throughput characterization of complex intact O-glycopeptide mixtures. J Am Soc Mass Spectrom.

[107] Lu L., Riley N.M., Shortreed M.R., Bertozzi C.R., Smith L.M. (2020). O-pair search with MetaMorpheus for O-glycopeptide characterization. bioRxiv.

[108] Polasky D.A., Yu F., Teo G.C., Nesvizhskii A.I. (2020). Fast and comprehensive N- and O-glycoproteomics analysis with MSFragger-glyco. bioRXiv.

[109] Hu H., Khatri K., Zaia J. (2017). Algorithms and design strategies towards automated glycoproteomics analysis: algorithms and design strategies. Mass Spectrom Rev.

[bib110] Abrahams J.L., Taherzadeh G., Jarvas G., Guttman A., Zhou Y., Campbell M.P. (2020). Recent advances in glycoinformatic platforms for glycomics and glycoproteomics. Curr Opin Struct Biol.

[111] Bennun S.V., Baycin Hizal D., Heffner K., Can O., Zhang H., Betenbaugh M.J. (2016). Systems glycobiology: integrating glycogenomics, glycoproteomics, glycomics, and other ‘omics data sets to characterize cellular glycosylation processes. J Mol Biol.

[112] Woo C.M., Felix A., Byrd W.E., Zuegel D.K., Ishihara M., Azadi P., Iavarone A.T., Pitter S.J., Bertozzi C.R. (2017). Development of IsoTaG, a chemical glycoproteomics technique for profiling intact N- and O-glycopeptides from whole cell proteomes. J Proteome Res.

[113] Qin K., Zhu Y., Qin W., Gao J., Shao X., Wang Y.L., Zhou W., Wang C., Chen X. (2018). Quantitative profiling of protein O-GlcNAcylation sites by an isotope-tagged cleavable linker. ACS Chem Biol.

[114] Plomp R., Bondt A., de Haan N., Rombouts Y., Wuhrer M. (2016). Recent advances in clinical glycoproteomics of immunoglobulins (igs). Mol Cell Proteomics.

[115] Alagesan K, Kolarich D: To enrich or not to enrich: enhancing (glyco)peptide ionization using the CaptiveSpray nanoBoosterTM. bioRxiv 597922, doi:10.1101/597922.

[bib116] Stavenhagen K., Kayili H.M., Holst S., Koeleman C.A.M., Engel R., Wouters D., Zeerleder S., Sajih B., Wuhrer M. (2018). N- and O -glycosylation analysis of human C1-inhibitor reveals extensive mucin-type O -glycosylation. Mol Cell Proteomics.

[117] Madsen J.A., Joon Ko B., Xu H., Iwashkiw J.A., Robotham S.A., Shaw J.B., Feldman M.F., Brodbelt J.S. (2013). Concurrent automated sequencing of the glycan and peptide portions of O -linked glycopeptide anions by ultraviolet photodissociation mass spectrometry. Anal Chem.

[118] Ko B.J., Brodbelt J.S. (2015). Comparison of glycopeptide fragmentation by collision induced dissociation and ultraviolet photodissociation. Int J Mass Spectrom.

[119] Gray C.J., Compagnon I., Flitsch S.L. (2020). Mass spectrometry hybridized with gas-phase InfraRed spectroscopy for glycan sequencing. Curr Opin Struct Biol.

[120] Schindler B., Barnes L., Gray C.J., Chambert S., Flitsch S.L., Oomens J., Daniel R., Allouche A.R., Compagnon I. (2017). IRMPD spectroscopy sheds new (infrared) light on the sulfate pattern of carbohydrates. J Phys Chem.

[121] Zhou C., Schulz B.L. (2020). Glycopeptide variable window SWATH for improved data independent acquisition glycoprotein analysis. Anal Biochem.

[122] Ye Z., Mao Y., Clausen H., Vakhrushev S.Y. (2019). Glyco-DIA: a method for quantitative O-glycoproteomics with in silico-boosted glycopeptide libraries. Nat Methods.

[123] Chen Z., Glover M.S., Li L. (2018). Recent advances in ion mobility–mass spectrometry for improved structural characterization of glycans and glycoconjugates. Curr Opin Chem Biol.

[124] Izaham A.R.A., Ang C., Nie S., Bird L.E., Williamson N.A., Scott N.E. (2020). What are we missing by using hydrophilic enrichment? Improving bacterial glycoproteome coverage using total proteome and FAIMS analysis. bioRxiv.

[125] Narimatsu H., Kaji H., Vakhrushev S.Y., Clausen H., Zhang H., Noro E., Togayachi A., Nagai-Okatani C., Kuno A., Zou X., Cheng L., Tao S.-C., Sun Y. (2018). Current technologies for complex glycoproteomics and their applications to biology/disease-driven glycoproteomics. J Proteome Res.

[126] Szychowski J., Mahdavi A., Hodas J.J.L., Bagert J.D., Ngo J.T., Landgraf P., Dieterich D.C., Schuman E.M., Tirrell D.A. (2010). Cleavable biotin probes for labeling of biomolecules via azide-alkyne cycloaddition. J Am Chem Soc.

[127] Miyamoto D.K., Flaxman H.A., Wu H.Y., Gao J., Woo C.M. (2019). Discovery of a Celecoxib Binding Site on Prostaglandin e Synthase (PTGES) with a Cleavable Chelation-Assisted Biotin Probe. ACS Chem Biol.

[bib128] Schjoldager K.T., Joshi H.J., Kong Y., Goth C.K., King S.L., Wandall H.H., Bennett E.P., Vakhrushev S.Y., Clausen H. (2015). Deconstruction of O-glycosylation-GalNAc-T isoforms direct distinct subsets of the O-glycoproteome. EMBO Rep.

[129] Fujitani N., Furukawa J. ichi, Araki K., Fujioka T., Takegawa Y., Piao J., Nishioka T., Tamura T., Nikaido T., Ito M. (2013). Total cellular glycomics allows characterizing cells and streamlining the discovery process for cellular biomarkers. Proc Natl Acad Sci.

[bib130] Rivas M.D. Las, Lira-Navarrete E., Daniel E.J.P., Companõn I., Coelho H., Diniz A., Jiménez-Barbero J., Peregrina J.M., Clausen H., Corzana F. (2017). The interdomain flexible linker of the polypeptide GalNAc transferases dictates their long-range glycosylation preferences. Nat Commun.

[bib131] De Las Rivas M., Paul Daniel E.J., Coelho H., Lira-Navarrete E., Raich L., Compañón I., Diniz A., Lagartera L., Jiménez-Barbero J., Clausen H. (2018). Structural and mechanistic insights into the catalytic-domain-mediated short-range glycosylation preferences of GalNAc-T4. ACS Cent Sci.

[bib132] de las Rivas M., Paul Daniel E.J., Narimatsu Y., Compañón I., Kato K., Hermosilla P., Thureau A., Ceballos-Laita L., Coelho H., Bernadó P. (2020). Molecular basis for fibroblast growth factor 23 O-glycosylation by GalNAc-T3. Nat Chem Biol.

[bib133] Fernandez A.J., Daniel E.J.P., Mahajan S.P., Gray J.J., Gerken T.A., Tabak L.A., Samara N.L. (2019). The structure of the colorectal cancer-associated enzyme GalNAc-T12 reveals how nonconserved residues dictate its function. Proc Natl Acad Sci.

[134] Zol-Hanlon M.I., Schumann B. (2020). Open questions in chemical glycobiology. Commun Chem.

[135] Mahal L.K., Yarema K.J., Bertozzi C.R. (1997). Engineering chemical reactivity on cell surfaces through oligosaccharide biosynthesis. Science.

